# AQUACULTURE: A Second Look at Sea Lice

**DOI:** 10.1289/ehp.119-a69

**Published:** 2011-02

**Authors:** David A. Taylor

**Affiliations:** **David A. Taylor** writes for *The Washington Post* and *Smithsonian* and is author of *Ginseng, the Divine Root*, about the science and subculture surrounding the medicinal plant. He teaches science writing at The Writer’s Center in Maryland

A new study has reopened an old debate over the potential health risks that fish farms pose to wild fish populations, concluding that “productivity of wild salmon is not negatively associated with either farm lice numbers or farm fish production.”^1^ The paper by Gary Marty, a research associate at the University of California, Davis, and fish pathologist with the British Columbia Ministry of Agriculture, and two colleagues pooled data from fish farms in western Canada with data first presented in 2007 by Krkošek et al.^2^ The earlier paper concluded that infestations of ectoparasitic sea lice from salmon farms were driving a decline in wild pink salmon (*Oncorhynchus gorbuscha*) populations in British Columbia’s Broughton Archipelago and that extinction would occur if the infestations continued.

Sea lice pose no direct threat to humans who consume the fish; furthermore they’re removed during the harvesting process. But the new study^1^ contributes to the larger ongoing discussion of whether a large-scale aquaculture industry can be sustainable in terms of human and ecosystem health.

Marty says earlier analyses omitted relevant factors from a medical perspective—that is, a diagnostic approach to fish health and epidemiologic factors, rather than a model-driven analysis. For the new study, he and his colleagues obtained proprietary monthly sea lice data from fish farms in the region, giving what they call a fuller picture of the salmon decline in 2001–2002 than the previous analyses, which relied on sea lice counts from wild fish only.

According to Marty, the new analysis suggests pink salmon populations are within a natural pattern of fluctuation. “Our paper estimates that sea lice numbers on farmed fish were greater in 2000 than in 2001, and the wild pink salmon exposed to those sea lice in 2000 came back in record high numbers in 2001,” he says.

Martin Krkošek, lead author of the 2007 paper^2^ and a lecturer in zoology at the University of Otago in Dunedin, New Zealand, says the new analysis was limited by the omission of data from the affected region prior to infestations as well as nearby regions where there are no salmon farms. Analyses that used the spatial and temporal controls from a larger picture of salmon abundance in the Broughton Archipelago, Krkošek says, “have found effects of sea lice.”^3^,^4^

Jeff Silverstein, national aquaculture program leader for the U.S. Department of Agriculture, notes that although the earlier paper^2^ suggested sea lice from salmon farms caused wild salmon declines, “The recent study^1^ has managed to show that the correlations don’t appear to be causative.” Ian Bricknell, director of the Aquaculture Research Institute at the University of Maine in Orono, adds, “The epidemiological approach . . . is a much more effective way of analyzing this data.” Bricknell says Marty et al. “looked at many more variables than just lice and salmon (as was done earlier) and have backed it up by testing their model with biological data.”

Krkošek agrees other factors may have contributed to the 2002 decline but strongly disagrees with the conclusion that sea lice do not negatively affect wild salmon productivity.^4^ “While one may speculate about the possibility of other factors that could have contributed, it is known—not speculated—that lice numbers were very high on those fish,” he says.

Marty insists the study’s most important impact lies in showing how medical analysis brings a broader perspective to fish population studies. “I want people to focus on what is actually causing salmon populations to go up and down,” he says. “We should still look at sea lice but include other factors as well.”
